# Reward modulation of contextual cueing: Repeated context overshadows repeated target location

**DOI:** 10.3758/s13414-017-1397-3

**Published:** 2017-08-07

**Authors:** Fariba Sharifian, Oliver Contier, Claudia Preuschhof, Stefan Pollmann

**Affiliations:** 1 Department of Experimental Psychology, Otto-von-Guericke University, 39106 Magdeburg, Germany; 2 Department of Experimental Psychology, Institut für Psychologie II, Otto-von-Guericke-Universität Magdeburg, Universitätsplatz 2, Gebäude 24, 39106 Magdeburg, Germany; 3 Center for Behavioral Brain Sciences, 39106 Magdeburg, Germany

**Keywords:** Visual search, Implicit/explicit memory

## Abstract

Contextual cueing can be enhanced by reward. However, there is a debate if reward is associated with the repeated target–distractor configurations or with the repeated target locations that occur in both repeated and new displays. Based on neuroimaging evidence, we hypothesized that reward becomes associated with the target location only in new displays, but not in repeated displays, where the repeated target location is overshadowed by the more salient repeated target–distractor configuration. To test this hypothesis, we varied the reward value associated with the same target location in repeated and new displays. The results confirmed the overshadowing hypothesis in that search facilitation in repeated target–distractor configurations was modulated by the variable value associated with the target location. This effect was observed mainly in early learning.

Visual search can be guided by memory when we encounter the same visuospatial configurations repeatedly. This effect, which occurs even in random meaningless configurations and in the absence of learning instructions, has been termed contextual cueing (Chun & Jiang, 1998).

Contextual cueing can be modulated by reward. Tseng and Lleras (2013) showed that repeated displays were learned faster when consistently paired with high reward. However, Schlagbauer, Geyer, Muller, and Zehetleitner (2014) pointed out that not only the distractor configuration is repeated in the contextual-cueing paradigm but also the target location, in repeated displays as well as in new displays. This is done to deconfound learning effects due to distractor configuration learning from those due to target-location probability learning (Geng & Behrmann, 2005; Jiang, Swallow, Rosenbaum, & Herzig, 2013; J. Miller, 1988). However, when reward is consistently paired with repeated displays, it is also paired with the repeated target location. To investigate effects of reward on contextual cueing and on target probability learning, Schlagbauer et al. (2014) crossed reward magnitude in a full factorial design with configuration, so that one half of repeated and new configurations were paired with either high or low reward. They found that search times were reduced in new displays associated with high reward, indicating that reward value was associated with the target location in the absence of repeated configurations. In a further study that had an analogous design to the experiment by Schlagbauer et al. (2014) but additionally used functional magnetic resonance imaging (fMRI) to investigate the involvement of dopaminergic brain structures in reward modulation, reward value modulated ventral striatal activation in new displays but not in repeated displays (Pollmann, Estocinova, Sommer, Chelazzi, & Zinke, 2016). This study consisted of a behavioral training session and a subsequent fMRI session. During the training session, high or low monetary rewards were associated with distinct sets of repeated displays as well as distinct sets of repeated target locations in new displays. In the fMRI experiment, the same displays were presented, but no rewards could be earned, so that activation changes between the high versus low reward displays could only be due to an association of reward with either context or target location that had been learned in the training session. In addition to activation changes related to reward modulation of contextual cueing, we found a modulation of activity in the ventral striatum by reward only for new displays. As only the target location but not the context was repeated in new displays, the observed modulation had to be due to an association of reward value with target location. However, if the reward modulation in the ventral striatum was due to reward association with the target location, then why was no such reward modulation observed for repeated displays, in which the target location was repeated in the same way as in new displays? A possible explanation could be that learning a reward association of a repeated target location is overshadowed when the context is also repeated in a display, so that the target value becomes associated with the context but not (or much less) with the target location.

## The overshadowing hypothesis

In classical conditioning, overshadowing describes the effect that two conditioned stimuli that are presented contingent with a reward compete for reward association and one of the stimuli, typically the more salient one, may become associated with the reward whereas the other stimulus is not or much less associated with the reward (Pavlov, 1927). Overshadowing is similar to the blocking effect (Kamin, 1969), in which a second stimulus that is added to an already conditioned stimulus is not learned if it has the same contingency with the reward (i.e., carries no additional information for reward prediction). This overshadowing hypothesis was investigated with respect to the contextual-cueing paradigm in the present study. Specifically, we expected that target location and distractor context will compete for reward association, when both are consistently associated with a reward value. That is, in a display in which context and target location are repeatedly presented, followed by the same reward value, the reward could be associated to the target–distractor configuration on the one hand or solely to the target location on the other hand. Moreover, we expected that the more salient repeated context will “win” this competition after several repetitions. In contrast, in new displays, where only the target location is repeated, consistent pairing with reward value was expected to lead to an association of reward value and target location. Crucially, when both repeated and new displays share the same target location, target-location probability cueing and contextual cueing will compete, slowing down the search facilitation observed for repeated displays due to contextual cueing. This competition between target-location probability cueing and contextual cueing might be increased for high rewards compared with low rewards associated with the same target location in new and repeated rewards. When a target location is paired with the same (high or low) reward value in repeated and new displays, no clear prediction can be made if this will benefit contextual cueing, target-location probability cueing, or both. However, the overshadowing hypothesis can be tested by varying the reward value associated with the same target location in new and repeated displays. When the same target location is paired with high reward value in new displays and with low reward value in repeated displays, target-location probability cueing should interfere more strongly with contextual cueing than in the reverse case (i.e., low reward for new and high reward for repeated displays).

Please note that the overshadowing hypothesis relates specifically to the association of reward value with target location or context. While no evidence for cue competition—as in overshadowing or the related blocking phenomenon—was observed related to contextual cueing itself (Beesley & Shanks, 2012), evidence for Pavlovian reward learning was reported in a value-driven attentional capture paradigm (Bucker & Theeuwes, 2017). Thus, it may be that learning of search contexts or target-location probability may not be governed by classical conditioning principles (but see Jiang & Leung, 2005, for contrasting evidence), whereas reward modulation of these learning processes does so.

**Fig. 1:**
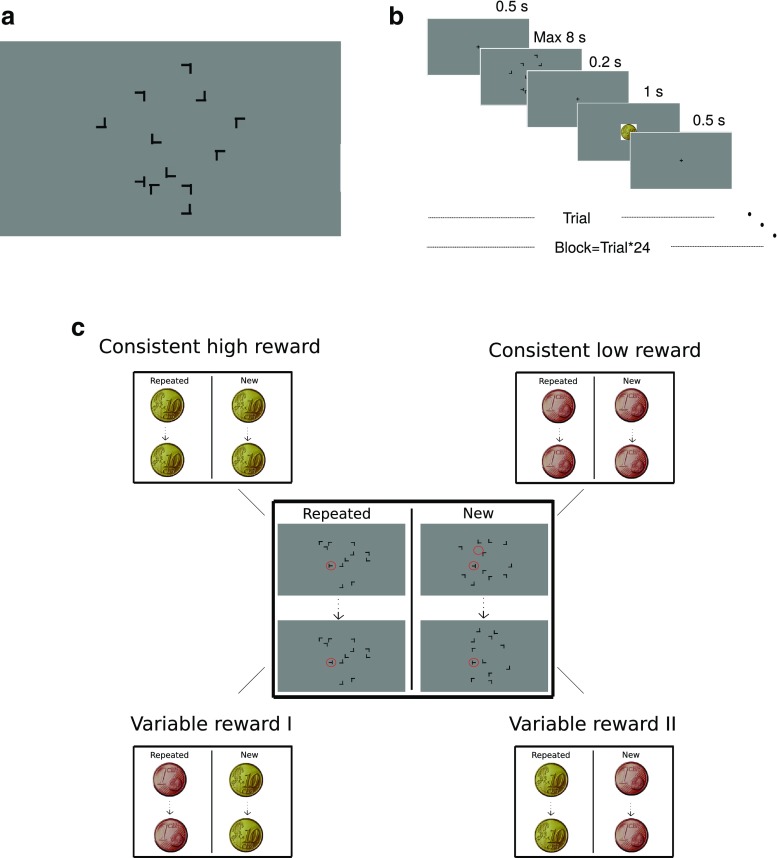
Stimuli and experimental design. **a** Scaled screenshot of one sample search display with one target (T-shaped item) and 11 distractors (L-shaped items). **b** Timing of a trial. **c** Reward allocation to target locations were divided into four different categories: consistent high reward, consistent low reward, variable reward with low reward in repeated displays and with high reward in new displays (Variable Reward I) and vice versa (Variable Reward II). (Color figure online)

To test the overshadowing hypothesis, we disambiguated reward modulation of contextual cueing versus target-location probability cueing. The same set of 12 target locations was used for repeated and new displays. Half of the target locations were consistently paired with either high or low reward. The other half of the target locations switched reward value between repeated and new displays, so that target locations associated with high reward in repeated displays were paired with low reward in new displays and vice versa (see Fig. 1c).

## Method

### Participants

Fifty-one young volunteers (22 males; age *M* = 23.6, *SD* = 3.5 years, range: 19–33 years) participated in the experiment. All the participants had normal or corrected-to-normal vision without any recognized neurological disorder history. The subjects were naïve to the purpose of the present research. Immediately after the experiment, the participants received a fixed reimbursement (€6 for each hour) plus a variable pecuniary reward dependent on their performance in the experiment task (*M* = 19.2, *SD* = €1.3, range: €16–21). Two subjects (one male and one female, age 33 and 20 years, respectively) were excluded from the analysis due to poor task performance (less than 90% correct answers; both received €16 rewards). The response accuracy for all the other subjects was on average 96.8% (*SD* = 2.4%). The experiments were approved by the ethics committee of the Otto-von-Guericke-University Magdeburg and carried out in agreement with the tenets of the Declaration of Helsinki. All the subjects provided written informed consent for taking part in this study prior to starting the experiments.

### Stimuli

The stimuli were created and presented with the Psychophysics Toolbox Version 3 (Brainard, 1997) MATLAB (MathWorks, Sherborn, MA) toolbox. The stimuli were displayed on an MS-Windows machine on a screen with 1920 × 1200 pixels resolution and 60 Hz refreshing rate. The viewing distance was fixed for all the subjects to 57 cm by means of a chin rest.

Each display (see Fig. 1a) comprised 11 L-shaped and one T-shaped black 1.25° × 1.25° items that were presented on a gray (#808080) background. The only T-shaped item in each display was the target, and it had a 90° clockwise (termed left) or counterclockwise (termed right) rotation; the subjects were instructed to discriminate the right or left rotation of the target. The number of rotations of target were equally often to the left and right. The L-shaped distractors were randomly rotated by 0°, 90°, 180°, or 270°. In order to increase the task difficulty (Jiang & Chun, 2001), the line junction of the L-shaped items had 4 pixels offset to make them similar to the T-shaped target. The items could take place on imaginary circles around central fixation with a 2.0°, 4.6°, 7.3°, and 10.0° radius and with four, 12, 20, and 28 possible locations in each eccentricity, respectively. Twelve locations with 4.6° and 7.3° eccentricity were randomly selected as potential target locations. Targets locations in the practice block were chosen from the remaining locations (after selecting training target locations) at 4.6° and 7.3° eccentricities. The other locations, which were not chosen as training or practice target locations, were used as potential distractor locations in the practice block. In each display, all the items were balanced across display quadrants. This randomization was done for each subject separately.

### Experimental design

The study was conducted in a sound-attenuated testing chamber. Each trial began with 0.5 s presentation of a fixation cross, followed by presentation of the search display until the subject’s manual response or a maximum of 8 s. This was followed by a 0.2-s blank interval, a 1-s presentation of feedback to the manual response, and a 0.5-s blank intertrial interval. Participants were instructed to report the orientation of the target (“T” stem pointing to the left or right) by pressing the 1 or 2 key on a standard keyboard number pad, respectively. The participants were instructed to respond as fast and accurately as possible. In the feedback phase of each trial, a coin (size: 9.1° diameter) representing the reward value was presented if a correct response was received within a time window of 2.5 s from display onset. The reward could be either 10 cents euro or 1 cent euro, termed *high* and *low* rewards, respectively. The feedback for trials with incorrect answer or response times longer than 2.5 s was a dark gray circle (size: 9.1° diameter) plus an additional 0.5 s of a low-pitch tone (300 Hz) for incorrect answers.

In addition to a practice block that was not further analyzed, the experiment consisted of 16 training blocks. In the practice block, the subjects practiced the task in 24 trials without receiving or showing any reward for their responses. Each following block also consisted of 24 trials (see Fig. 1b). The sum of the rewards and the average response time for each block were reported to the subjects at the end of that block and the subjects were allowed to take breaks between the blocks.

For each subject, 12 positions in the second or third imaginary rings were randomly selected as target locations. There were same numbers of target locations in each quadrant, and in each quadrant, the number of target locations was proportionally distributed among the imaginary rings based on the available positions in that ring. In each block, each target position was presented once in a repeated and once in a new display. In repeated displays, the position and orientation of the distractors were kept constant along with the target location, whereas both were randomly changed in new displays. Target orientation was varied randomly in both new and repeated displays so that no association could be learned between repeated configurations, target locations, or reward value with a particular response.

The 12 target locations were divided into four different categories: (1) Three target locations were consistently combined with high reward (10 cents euro) for both repeated and new displays; (2) three more target locations were combined with low reward (1 cent euro) in both repeated and new displays; and Of the remaining six target locations, (3) three were combined with low reward in repeated displays and with high reward in new displays, and the remaining (4) three target locations were associated with high reward in repeated displays and with low reward in new displays (see Fig. 1c). In this way, we orthogonally varied reward association with target–distractor configuration (repeated, new) and with target location (consistent, variable).

### Analysis

Search time was the primary dependent variable. Only trials with correct responses were analyzed. We averaged the results of every four successive blocks as one epoch. Effect size was measured as partial eta squared, respectively, Cohen’s *d* value.

## Results

A repeated-measures analysis of variance (ANOVA) was calculated on search times, with the factors epoch (1–4), configuration (new, repeated), reward value (high, low), and consistency (consistent, variable).

**Fig. 2:**
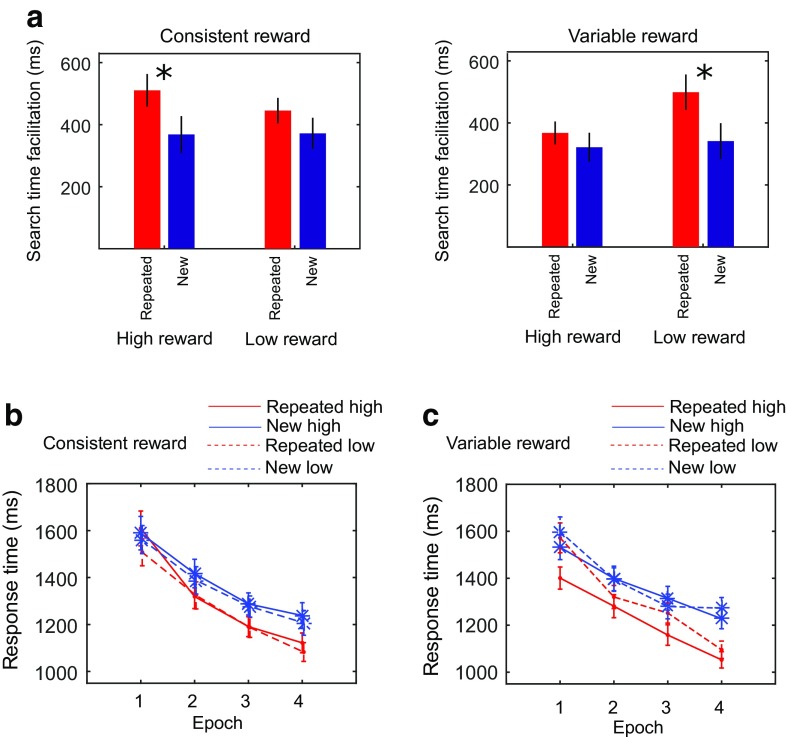
Search time modulation. **a** Search time reduction from Epoch 1 to Epoch 4 for the consistent reward and variable reward conditions. **b** Average search time in ms for all epochs across all subjects in repeated (*red line*) and new (*blue line*) configurations for consistent high and low reward, and **c** variable high and low reward. In the consistent reward condition, repeated low (high) reward displays share the same target locations with new low (high) reward displays. In the variable reward condition, repeated low (high) reward displays share the target location with new high (low) reward displays. *Error bars* show standard errors of the means and asterisks indicate statistical significance. (Color figure online)

The ANOVA revealed significant main effects of epoch, *F*(1, 48) = 242.38, *p* < .05, η_p_^2^ = 0.83, and configuration, *F*(1, 48) = 34.89, *p* < .05, η_p_^2^ = 0.42, and a significant Epoch × Configuration interaction, *F*(1, 48) = 11.06, *p* < .05, η_p_^2^ = 0.19. In line with the expected contextual-cueing effect, search times decreased over the course of the experiment, particularly for repeated displays (see Fig. 2). Of particular importance for the overshadowing hypothesis, the Reward × Consistency interaction, *F*(1, 48) = 6.36, *p* < .05, η_p_^2^ = 0.12, was also significant, as was the Epoch × Reward × Consistency interaction, *F*(1, 48) = 4.59, *p* < .05, η_p_^2^ = 0.09, and the four-way interaction, *F*(1, 48) = 1102.03, *p* < .05, η_p_^2^ = 0.96; all other effects *F*(1, 48) < 3.13, *p* > .08, η_p_^2^ < 0.06.

Figure 2a shows that the four-way interaction was due to stronger search facilitation (reduced search times) from Epoch 1 to Epoch 4 for repeated, consistent high-reward displays and for repeated-variable low-reward displays. We used one-tailed paired-samples *t* tests to compare the search-time facilitation in new and repeated configurations. In consistent trials, high reward yielded a significantly larger search facilitation for repeated displays compared to new displays, *t*(48) = 2.11, *p* = .04, *d*= 0.38, whereas this was not observed for low reward, *t*(48) = 1.63, *p* = .11, *d* = 0.24. In contrast, in displays with variable target value, significant difference of search facilitation was not observed for repeated versus new high-reward displays, *t*(48) = 0.86, *p* = .38, *d* = 0.17, but was observed for repeated versus new low-reward displays, *t*(48) = 2.12, *p* = .04, *d* = 0.41.

While Fig. 2a allows a comparison with previous work, a more detailed look at the search-time pattern shows that it is somewhat misleading, in that lower search-time reductions from Epoch 1 to Epoch 4 are actually due to faster search times in Epoch 1. We had hypothesized that target-location probability cueing will interfere with the development of contextual cueing, an effect that should be present most strongly in the early display presentations, before contextual cueing finally becomes dominant. Therefore, we analyzed the first and the last epoch separately. As Fig. 2b–c show, there appeared to be search-time differences already in Epoch 1. The ANOVA on Epoch 1 showed a significant main effect of configuration, *F*(1, 48) = 4.07, *p* < .05, η_p_^2^ = 7.82, and a significant Consistency × Reward interaction, *F*(1, 48) = 7.66, *p* < .05, η_p_^2^ = 13.75; all other effects, *F*(1, 48) < 2.23, *p* > .14, η_p_^2^ < 4.44. In the consistent reward condition, repeated low-reward displays (sharing the target locations with new low-reward displays) were searched faster in absolute terms than repeated high-reward displays were (see Fig. 2b; 1,507 ms vs 1,609 ms). This difference, however, was not statistically significant, *t*(48) = 1.32, *p* = .19. Conversely, in the variable reward condition, where high-reward value in new displays was hypothesized to interfere with contextual cueing most strongly, indeed, repeated low-reward displays (sharing the target location with new high-reward displays) were slower than repeated high-reward displays (sharing the target locations with new low-reward displays) in Epoch 1 (see Fig. 2c; 1,401 ms vs. 1,573 ms), *t*(48) = 2.78, *p* < .05. In contrast, the ANOVA on Epoch 4 showed only a significant effect of configuration, *F*(1, 48) = 49.39, *p* < .05, η_p_^2^ = 50.72; all other effects and interactions, (1, 48) < 2.86, *p* > .10, η_p_^2^ < 5.61.

In addition, considering accuracy as the effect of interest an ANOVA analogous to the overall analysis of search times showed significant main effects of epoch, *F*(1, 48) = 8.11, *p* < .05, η_p_^2^ = 0.14, and configuration, *F*(1, 48) = 10.31, *p* < .05, η_p_^2^ = 0.18, as well as the four-way interaction, *F*(1, 48) = 39961.28, *p* < .05, η_p_^2^ = 1; all other effects, *F*(1, 48) < 3.69, *p* > .06, η_p_^2^ < 0.07. The average accuracy increased from average 95.9% (*SD* = 3.3%) in Epoch 1, to 97.1% (*SD* = 3.2%) in Epoch 4. Further, the average accuracy was slightly higher in repeated (*M* = 97.3%, *SD*= 2.1%) versus new (*M* = 96.3%, *SD* = 3.0%) configurations. Thus, there was no indication of a speed–accuracy trade-off.

## Discussion

Contextual cueing of visual search can be modulated by reward. However, there is a debate if reward is associated with repeated target–distractor configurations (Tseng & Lleras, 2013) or with repeated target locations (Schlagbauer et al., 2014). In the contextual-cueing paradigm, the probability of target repetition at a given location is held constant in both repeated and new context displays to deconfound context learning from target-location probability learning. When reward learning is paired with the contextual-cueing paradigm, the reward value can theoretically be associated with the repeated target-context configuration as well as with the repeated target locations, the latter in both repeated and new displays (Schlagbauer et al., 2014). Previous behavioral evidence was inconclusive with respect to these associations. Tseng and Lleras (2013) did not analyze reward association with target location in new displays. When this was done in the study by Schlagbauer et al. (2014), reward modulation of target location probability cueing was observed. In a previous study from our lab, we observed increased activation of the ventral striatum, a key region of the dopaminergic system involved in reward learning (Daniel & Pollmann, 2014) by new displays but not by repeated displays associated with high reward. Since only the target location was repeated—and could be learned—in new displays, we attributed this effect to a learned association of reward magnitude with the target location. However, it was puzzling that no activation difference was observed in repeated high-reward versus low-reward displays, in which the target-location probability and its associated reward presentation was the same as in the new displays. This led to the hypothesis tested in the present experiment, namely, that the association of reward magnitude with target location may be overshadowed by learning the context–reward association in repeated configurations.

In order to test this hypothesis, we disambiguated reward association with context and with target location. To this end, the same set of target locations was used in repeated and new displays. In half of the trials, the target location was consistently associated with either high or low reward across new and repeated displays. In the other half of trials, however, reward magnitude for the target location switched between repeated and new displays. In the consistent reward trials, we replicated previous findings in that high reward boosted search facilitation in repeated displays (Pollmann et al., 2016; Tseng & Lleras, 2013). In the variable reward trials, however, we observed—as predicted by the overshadowing hypothesis—increased interference of target location probability cueing with contextual cueing when the same target location was paired with high reward in new displays and low reward in repeated displays than vice versa. This shows that reward modulates target location probability learning more in new displays, in the absence of a repeated context, than in repeated displays. Interference of target location probability cueing and contextual cueing was observed early in learning, while it was absent at the end of the Experiment. This was expected, as in overshadowing, a small initial advantage in association strength of one stimulus compared with its competitor quickly leads to a massive dominance after several repetitions (R. R. Miller et al., 1995). Moreover, it is well known that contextual cueing can be observed already after a few repetitions (Tseng & Lleras, 2013).

Could the short search times for variable high-reward repeated displays in Epoch 1 be an artefact caused by idiosyncratic differences in the displays? We think this can be ruled out because displays were individually generated for each subject, and the large sample size makes between-condition differences in search difficulty based on display structure very unlikely.

Thus, reward modulates learning of a repeated target location or repeated context early on. This occurs in a competitive manner. Repeated context overshadows reward association with a repeated target location. Conversely, reward association with a repeated target location—learned in new displays—slows down learning of a repeated context. The latter is most prominent when the target location to be associated with a repeated context is associated with high reward in new displays.

On first sight, it may seem puzzling that we found clear evidence for target location value interference with contextual cueing but no direct evidence of faster search in new displays with high- versus low-reward target locations. Thus, reward association with the target location interfered with context learning or reward–context association, but it did not facilitate search in new displays. However, reward modulation of target-location probability cueing can only lead to a search-time advantage when high-reward targets are presented in some locations (e.g., in the upper left quadrant) more often than in others (Geng & Behrmann, 2005). In this case, preferential search in the high-reward target locations (the upper left quadrant) would lead to shorter response times to the high-reward targets. In our case, however, high- and low-reward targets were equally distributed over the display so that no search preference for a particular part of the display was expected to develop. The lack of a reward effect on new trial search times is consistent with a previous report from our lab that used a similar display layout (Pollmann et al., 2016). However, reward value modulated the search times for new displays in the study by Schlagbauer et al. (2014). These discrepant results may be due to differences in the display layout (e.g., all targets were arranged equidistant to the center in the Schlagbauer et al. study, but placed on two imaginary concentric circles by Pollmann et al., 2016, and in the current experiment) that did or did not enable participants to develop search biases that were specific enough to benefit high-reward target locations. In future experiments, high- versus low-reward target locations may be spatially arranged in such a way as to allow clear search benefits for high-reward target locations (e.g., Geng & Behrmann, 2005; J. Miller, 1988).

This also explains why reward modulation of the target location primarily interfered with the speed with which contextual cueing developed. In Epoch 4, after extensive learning, the search-time reduction for repeated over new displays was comparable for high- and low-reward displays, both in the consistent and variable reward conditions. Thus, it appears that reward modulates both target location probability learning and context learning early on, in an interactive manner, but affects search times less the more repetitions occur.

Please note that the results of the present study cannot directly be compared with the results of previous studies (Schlagbauer et al., 2014; Tseng & Lleras, 2013), because, in contrast to the present study, repeated and new conditions had separate sets of target locations, and, therefore, no direct carryover effects of target-location probability learning on contextual cueing or vice versa could occur. Nevertheless, overshadowing may explain a counterintuitive finding by Schlagbauer et al. (2014). They found stronger contextual cueing for low-reward trials than for high-reward trials. Contextual cueing was calculated by subtracting search times for old displays from search times for new displays. According to the overshadowing hypothesis, search facilitation by reward association with the target location would be stronger in new high-reward displays than in new low-reward displays, because there was no overshadowing by context in new displays (the search-time difference was indeed significant), whereas no differential search facilitation between high- and low-reward target value would occur in repeated displays (which were not significantly different), due to overshadowing by the repeated context. The difference between new and repeated search times thus should be smaller in high-reward displays than in low-reward displays, as observed by Schlagbauer et al. (2014).

In addition, our results fit into the context of priming in visual search (for a review, see Kristjansson, 2008), in which a priming effect was found not only for target repetition but also for distractor repetition (Wang, Kristjansson, & Nakayama, 2005), and this priming can be modulated by reward probability as well (Kristjansson, Sigurjonsdottir, & Driver, 2010).

In summary, our data show that the reward modulation of contextual cueing is influenced by an association of reward with repeated target–distractor configurations as well as with repeated target locations. However, the specific pattern that we observed shows that reward association of the target-location probability is dominantly learned in new displays, but overshadowed in the presence of repeated configurations.

(The code and data are available at https://osf.io/m6v5e/files/.)
